# Stochastic quantum Zeno-based detection of noise correlations

**DOI:** 10.1038/srep38650

**Published:** 2016-12-12

**Authors:** Matthias M. Müller, Stefano Gherardini, Filippo Caruso

**Affiliations:** 1 Department of Physics and Astronomy, LENS, QSTAR, University of Florence, via G. Sansone 1, I-50019 Sesto Fiorentino, Italy; 2 Department of Information Engineering, University of Florence, via S. Marta 3, I-50139 Florence, Italy; 3 CSDC, University of Florence, and INFN, via G. Sansone 1, I-50019 Sesto Fiorentino, Italy

## Abstract

A system under constant observation is practically freezed to the measurement subspace. If the system driving is a random classical field, the survival probability of the system in the subspace becomes a random variable described by the Stochastic Quantum Zeno Dynamics (SQZD) formalism. Here, we study the time and ensemble average of this random survival probability and demonstrate how time correlations in the noisy environment determine whether the two averages do coincide or not. These environment time correlations can potentially generate non-Markovian dynamics of the quantum system depending on the structure and energy scale of the system Hamiltonian. We thus propose a way to detect time correlations of the environment by coupling a quantum probe system to it and observing the survival probability of the quantum probe in a measurement subspace. This will further contribute to the development of new schemes for quantum sensing technologies, where nanodevices may be exploited to image external structures or biological molecules via the surface field they generate.

The dynamical evolution of a quantum system is always influenced by its environment^1^,^2^. Since one is very often only interested on the system dynamics, the environmental degrees of freedom are traced out and, in the Markovian regime (under the assumption of only very short-lived correlations), this leads to the well-known Kossakowski-Lindblad master equation^3^. As a consequence, this approximation does not take into account all the environment-induced memory effects, which may produce a back flow of information onto the quantum system^4^,^5^. However, the environment is usually unknown and very hard to be characterized. Therefore, in the last few years several theoretical proposals for the characterization of the environment according to whether it can generate Markovian or non-Markovian dynamics of the system to which it is coupled have been put forward, and a full hierarchy of non-Markovianity^6^ has been introduced. In particular, it has been shown that classical environments exhibiting time-correlated random fluctuations can lead to non-Markovian quantum dynamics^7^,^8^. The structure of such an environment can be probed by coupling a (typically small, e.g. one qubit) quantum system (probe) of known dynamics to it, and studying the effect of the environment on the system dynamics. Indeed, the very recent idea of the so-called quantum probes is that their fragile properties, as coherence and entanglement, are strongly affected by the environment features and can be used to detect them. Examples of such physical systems are quantum dots, atom chips and nitrogen vacancy centers in diamond where a good control over the system has been proposed and recently achieved^9^,^10^,^11^,^12^,^13^,^14^,^15^,^16^,^17^. They can be used to probe environments like biological molecules or surfaces of solid bodies or amorphous materials. In this regard, a number of non-Markovianity measures and witnesses has been proposed, such as geometric measures (i.e. measures based on the geometry of the space of quantum maps), quantities based on the Helstrom matrix (i.e. based on the distinguishability of two states under evolution and observation), or witnesses based on the (non-)monotonicity of entanglement measures^6^. Most of them, however, rely on a full state tomography and, thus, are experimentally difficult to be implemented. An experimentally feasible tool for certain systems is based on the state distinguishability and the Loschmidt echo^18^.

Recently, the scenario of stochastic measurement sequences has been proposed^19^, and then studied with a particular focus on the probability for the system (survival probability) to remain confined within a given measurement subspace^20^,^21^,^22^. Indeed, when the time interval between two measurements is random, this survival probability becomes a random variable by itself, and it has been shown by large deviation theory^23^,^24^,^25^ that it converges to its most probable value, by increasing the number of the measurements performed on the system^20^. When the measurements become very frequent, the survival probability increases and a stochastic quantum Zeno regime is accessed^20^,^26^. It is the stochastic generalization of quantum Zeno dynamics (QZD), where in the limit of infinitely frequent observation the dynamics of a quantum system is freezed to a unidimensional^27^ or multidimensional^28^,^29^ subspace of the measurement operator. QZD has been experimentally realized first with a rubidium Bose–Einstein condensate in a five-level Hilbert space^30^, and later in a multi-level Rydberg state structure^31^. Furthermore, a recent theoretical study and experimental demonstration with atom-chips has shown also how different statistical samplings of a randomly-distributed sequence of projective measurements coincides in the quantum Zeno regime, proving an ergodicity hypothesis for randomly perturbed quantum systems^21^. In this regard, the sensitivity of the survival probability to the stochasticity in the time interval between measurements has been properly analyzed by means of the Fisher information^22^.

In this work, we propose a method based on the Stochastic Quantum Zeno Dynamics (SQZD)^20^,^26^ to detect time correlations in random classical fields. Indeed, we use the SQZD formalism to study a quantum system (the probe), subjected to a sequence of equally spaced projective measurements, interacting with an environment modelled by a randomly fluctuating field. Then, the random value of the field leads to a random value of the survival probability in the measurement subspace. In this way, a witness for the environment time correlations can be obtained without characterizing the non-Markovianity of the quantum probe, by properly analyzing the behaviour of the time and ensemble average of this fluctuating survival probability with respect to the amount of noise temporal correlations. As outline, we first introduce our model of a quantum system coupled to the environment. Then, we review and adapt the SQZD formulation, and show how time correlations in the fluctuating field correspond to different statistical sampling of the random measurements. Finally, we demonstrate for random telegraph noise^32^,^33^ the imprint of the time scale of the correlated noise on the final survival probability after applying the entire measurement sequence.

## Model

### Stochastic Schrödinger Equation

**Figure 1:**
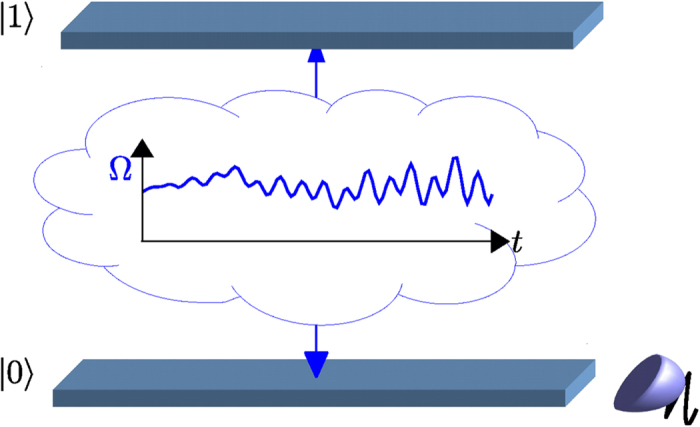
A random Hamiltonian driving couples the two levels |0〉 and |1〉 of a two-level quantum system. The system is initially prepared in |0〉. By measuring the remaining population in |0〉, we can extract information about the fluctuating field driving the system dynamics.

We study a quantum system that is coupled to a bath that effectively acts on the system via a time fluctuating classical field Ω(*t*) as


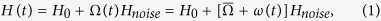


where *H*_0_ is the Hamiltonian of the unperturbed system, while *H*_*noise*_ describes the coupling of the environment to the system. In other words, we are modelling the classical environment as a random field represented by the stochastic process Ω(*t*). We assume that Ω(*t*) takes real values with mean 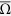, and *ω*(*t*) is the fluctuating part with vanishing mean value. Figure 1 shows an exemplary two-level system initially prepared in the ground state |0〉. The random Hamiltonian driving term causes a population transfer to the upper level |1〉. This can be probed by measuring the remaining population in |0〉. The system dynamics for a given realization of the random field Ω(*t*) is described by the standard Schrödinger equation. If we average over the statistics of the field Ω(*t*), we find the following master equation:


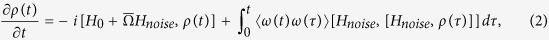


where 〈*ω*(*t*)*ω*(*τ*)〉 is the second-order time correlation of the random field, and [·, ·] represents the commutator. The reduced Planck’s constant *ħ* is set to unity. For white noise the second-order time correlation is a Dirac delta distribution, i.e. 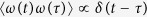, and we find the Lindblad-Kossakowski master equation^3^. In general, the memory kernel can lead to non-Markovian dynamics depending on the structure and time scale of the Hamiltonian, as for example demonstrated for random telegraph noise (RTN) and 1/*f*-noise^7^,^8^. Indeed, also a Markovian stochastic process Ω(*t*) (as in the case of RTN, where the time correlation is 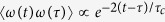) can lead to non-Markovian dynamics of the quantum system it is coupled to. In particular, in ref. 7 the quantum dynamics of the probe system, a qubit subject to RTN, can be Markovian or non-Markovian depending on the parameters of the non-fluctuating Hamiltonian term *H*_0_. This proves that Markovianity or non-Markovianity are not a feature of just the noise but of the dynamics of the system coupled to the noise. Thus, in this work we want to analyze just the time correlations of the environment, that are independent from the system coupled to it.

We now consider a system under sequential measurement where each measurement (with projection operator П) occurs after a fixed time interval *μ*. We call *q*(Ω) the single measurement quantum survival probability that will depend on the value of Ω during this time interval and, thus, be a random variable. We can now generalize the survival probability to the stochastic process as follows


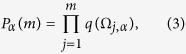


where *α* = 1, … *N* labels the realization of a trajectory, *j* represents the time order of the *m* measurements, and Ω_*j,*__*α*_(*t*) is the fluctuating field in this corresponding time interval. The single measurement quantum survival probability *q*(Ω_*j,*__*α*_), moreover, is defined as 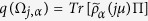, where 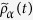 is the state of the system. In order to characterize the stochastic process *P*_*α*_(*m*), two natural quantities arise: the time average and the ensemble average of the survival probability. The time-average is defined here as


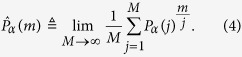


The idea is that, using the measured value of the survival probability after the *j*-th measurement, one can estimate the expectation value at *m* by 
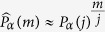
. We can then average this value for *j* = 1…*M* and take the limit of a large number of measurements *M*. This limit potentially depends on the realization *α* of the fluctuating field, as will be discussed below. The ensemble-average is instead defined as


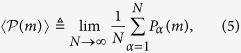


where the average of *P*_*α*_(*m*) is over a large number of realizations *N*. In the limit of infinite realizations, 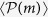 does not depend on the single realization but on their probability distribution. In the following section, we examine the behaviours of the time and ensemble averages of the survival probability *P*_*α*_(*m*), and in particular we study how correlations in the field fluctuations influence these averages.

## Results

For each realization *α* of the stochastic process we characterize the fluctuating field in between two measurements by a constant value Ω_*j*,*α*_(*t*) → Ω_*j*,*α*_ distributed according to a random distribution *p*(Ω). This is a valid formulation also for more complicated fluctuations, e.g. when the unitary dynamics is governed only by the fluctuating field, i.e. *H*_0_ = 0. In the latter case, the single quantum survival probabilities to survive in the initial state |*ψ*_0_〉, thus, become


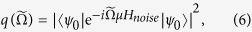


where 
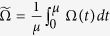
. Hence, *q* depends just on the constant 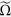 with *μ* being the length of the time interval between two measurements. Note that for simplicity we chose the initial state *ρ*_0_ = |*ψ*_0_〉〈*ψ*_0_| to be pure. However, the following main results depend just on the statistics of *q*(Ω) and not on the actual dependence of *q* on Ω. Thus, for non-vanishing *H*_0_ we treat Ω just as a parameter that describes the statistics of *q*(Ω) via the probability distribution *p*(Ω). Indeed, in the remainder of this section we do not specify *H*_0_, *H*_*noise*_, П, *ρ*_0_ or *μ* but use just *p*(Ω) and *q*(Ω).

**Figure 2:**
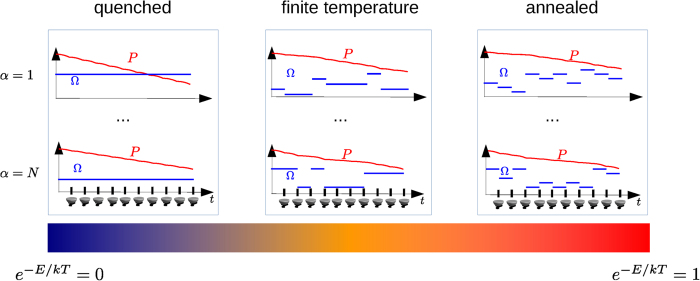
Schematic view of the field fluctuations and their influence on the survival probability *P* during the measurement sequence. The driving field Ω fluctuates in time and with increasing temperature the time correlations vanish, going from quenched disorder to annealed disorder. The survival probability *P* decreases in time at a rate depending on the average value of the fluctuating field. For annealed disorder the effect of the field fluctuations over the time intervals is averaged out and for each realization *P* converges almost to the same value. If we decrease the temperature the time correlation of the fluctuation grows and this convergence slows down. In the limit of *T* = 0 the fluctuations degenerate to a random offset value, determining the behavior of *P* that is now different for each realization.

Figure 2 shows in the right upper panel how the classical fluctuating field Ω causes the survival probability *P* to decrease at a fluctuating rate, such that a stronger average driving strength within one time interval causes a smaller *q* and a faster decrease of *P*. Within each time interval between two measurements the decrease of *P* is quadratic in the time interval and the field strength. While the field fluctuations are random (and independent identically distributed (i.i.d.)), after a few measurements the influence of these fluctuations on *P* are averaged out and the decay of *P* behaves similarly for each realization. When the field fluctuations are correlated, however, the decay of the survival probability depends much stronger on the realization because the probability distribution for Ω_*j*+1*,α*_ depends on the value of Ω_*j,α*_ (and potentially also on the previous history). This means that also the convergence of the time-average to its limit value is much slower, since a random deviation will influence not only a single time interval but a range of them, corresponding to the relaxation time *τ*_*c*_ associated to the time correlations. Now, we consider a simple correlation model inspired by random telegraph noise (RTN)^32^,^33^: we choose Ω_*j*+1*,α*_ according to the distribution *p*(Ω) only with a certain probability 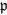, and Ω_*j*+1*,α*_ = Ω_*j,α*_ otherwise. This update probability 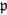 can be associated to a temperature *T* by 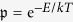 (see Fig. 2), and determines how strong the time correlations are. Indeed, the average time between two field switches is 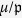, while for RTN this time equals the relaxation time *τ*_*c*_. The physical interpretation of this RTN environment is that the field value changes when, for example, a charge is trapped, and by thermal fluctuations a trapping energy barrier *E* has to be overcome for the charge to be released, such that the field value is restored to its previous value. In Fig. 2 temperature grows from left to right yielding different types of disorder. For *T* = 0, one has 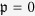, i.e. the value of the field Ω is chosen only once randomly and then always remains the same. The relaxation time *τ*_*c*_ is infinite and the time-average does not always converge to the same value. This scenario simulates the interaction of the system with an environment that exhibits *quenched* disorder. Depending on the value of Ω of the given realization *α*, the decay of the survival probability *P*_α_(*m*) can be faster or slower. On the other side, for infinite temperature we have 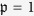, representing an *annealed* disorder environment. Between these two extreme regimes, i.e. for finite temperature, we have 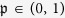, hence a mixture of both behaviours. Here, *quenched* disorder means a scenario with a static noise that depends on the initial random configuration of the environment, whereas *annealed* disorder means that the environment changes its configuration randomly in time^34^,^35^,^36^.

### Time and Ensemble Averages vs. Noise Correlations

For the time-average we introduce the expected frequencies *mn*_Ω_ with which the event Ω occurs in one realization of a stochastic sequence of *m* measurements. The time-average is then given by


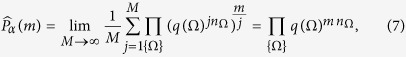


where the product is over all possible values of Ω and *n*_Ω_. For independent (thus uncorrelated) and identically distributed random variables Ω_*j,α*_ the expected frequencies correspond to the underlying probability distribution *n*_Ω_ = *p*(Ω). For correlated Ω_*j,α*_ the convergence of the time-average might not be unique or not even exist. This is linked to the Markov property and recurrence of the stochastic process^37^, as explained in more detail below by introducing the theoretical expressions for the time-average in different correlated dynamical regimes. Note that a Markovian stochastic process Ω(*t*) (or Ω_*j*_, with Ω_*j*_ depending just on Ω_*j*−1_ as in our model) does not imply Markovian quantum dynamics of the system since a Markovian fluctuating field can generate non-Markovian quantum dynamics through its time correlations^7^.

The ensemble average of the survival probability is the expectation value of the survival probability, i.e.


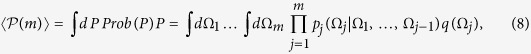


where 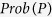 is the probability distribution of the survival probability *P*_*α*_(*m*) (which is by itself a random variable depending on the field fluctuations), and 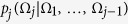 is the conditional probability for the event Ω_*j*_ given the process history. In the case of i.i.d. random variables Ω_*j*_, it becomes


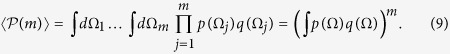


Now, we consider three different regimes to calculate the time and ensemble average by varying the value of 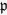: (1) annealed disorder (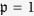), (2) a finite temperature case, with 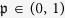 and the number of measurements *m* such that at least 5–10 jumps occur, and (3) quenched disorder (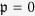). We introduce the shorthand notation 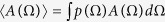, that we will use frequently for *A* = *q* and *A* = In *q*.

In the case of annealed disorder (*an*), i.e. uncorrelated noise, the theoretical expression for the two averages follows straightforwardly from the definitions, namely


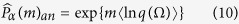


for the time-average and


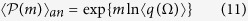


for the ensemble average – see Eq. (8). In the case of quenched disorder (*qu*), each single realization has constant *q*(Ω) and survival probability *q*(Ω)^*m*^. The ensemble average is, thus, the arithmetic average of these single possible outcomes, i.e.


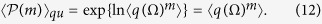


Instead, the time-average for quenched disorder does not take a single value but splits into several branches with


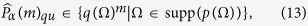


since the underlying process is not recurrent, in the sense that, given the value of Ω in the first interval, all the other values of the support of *p*(Ω) cannot be reached anymore within the given realization of the stochastic process.

Finally, for the finite temperature ( *fT* ) regime the problem is more difficult: the time-average is the same as for the annealed disorder, namely


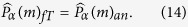


The reason is that, despite of the time correlations, after each field update event the field is chosen according to *p*(Ω) independently from the history of the process. Also 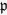 is independent of the current value of the field and for long times the frequencies of each *q*(Ω) still converge to their expected values *n*_Ω_ = *p*(Ω). Instead, in order to calculate the ensemble average we have to take into account all the time correlations examining the occurrence of sequences of Ω(*t*) that are constant over several time intervals and the updates of the probability according to 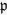. If the length of such a sequence is labelled by *k*, this length *k* is distributed by the Poisson distribution 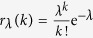 with 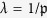 the inverse of the probability for an update of Ω. The survival probability for this sequence of constant field is:


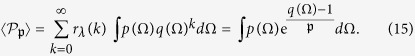


The frequency of the updates is also Poisson distributed, with expectation value 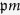. The joint survival probability is then


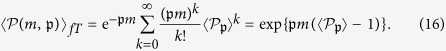


**Figure 3:**
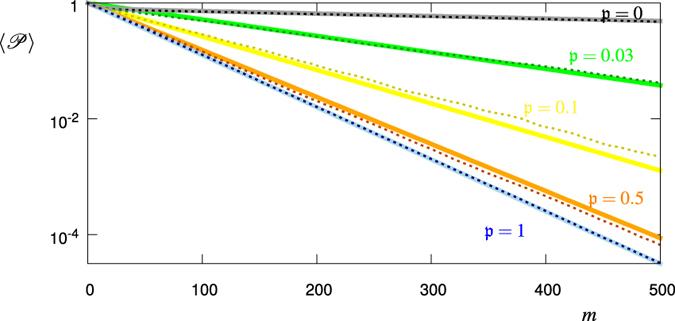
Ensemble Averages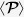, Eq. (5), for 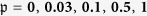 (black, green, yellow, red, blue). The dashed lines correspond to the values calculated from 1000 realizations of the stochastic process, while the solid lines correspond to the respective theory curves, Eqs. (11), (12), (16).

Figure 3 shows the above calculated ensemble averages together with numerical values from the realization of *N* = 1000 stochastic processes for different values of 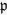. In all cases for *p*(Ω) we have used a bimodal distribution with *p*_1_ = 0.8, *p*_2_ = 0.2 and corresponding single measurement quantum survival probabilities *q*_1_ = 0.999, *q*_2_ = 0.9. If we decrease (increase) *q*_1_ and *q*_2_, the decay becomes faster (slower). The same happens for an increase (decrease) of *p*_2_, that is the probability associated with *q*_2_ < *q*_1_. Note that the probabilities *p*_1_, *p*_2_, *q*_1_ and *q*_2_ along with 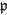 do fully define the above listed averages and we do not have to specify the Hamiltonian *H*(*t*). Their values in Fig. 3 have been chosen to model a system in the Zeno regime (*q*_1_, *q*_2_ close to 1) and with having in mind a bimodal noise field leading to two different possible values of *q*.

### Variance of the Survival probability

The variance and standard deviation of the the distribution 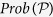 of the survival probability are defined as


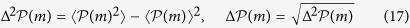


In order to calculate the variance, we still need to calculate the second moment of the probability distribution of the survival probability. In the special case of infinite temperature or annealed disorder, it is given by


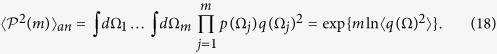


The normalized variance, thus, reads


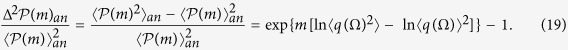


For finite temperature, we first consider again a sequence of constant Ω, where the square of the survival probability is as follows:


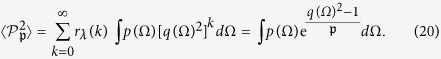


The frequency of the updates of the fluctuation field is again Poisson distributed, with expectation value 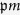. Then, the joint squared survival probability is


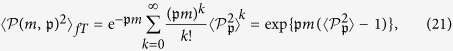


and the normalized variance reads


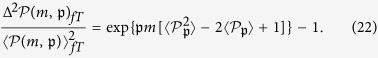


Finally, for quenched disorder one has


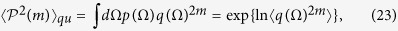


with the normalized variance


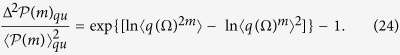


**Figure 4:**
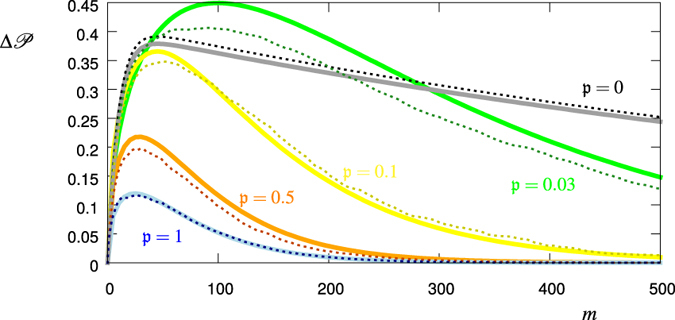
Standard deviation 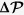, Eq. (17), for 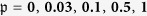 (black, green, yellow, red, blue). The dashed lines correspond to the value calculated from 1000 realizations of the stochastic process, while the solid lines correspond to the respective theory curve, Eqs. (18), (21), (23).

**Figure 5:**
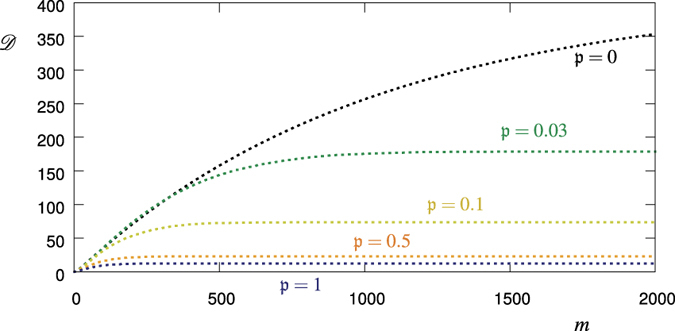
Accumulated standard deviation 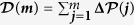, Eq. (25), as a function of *m* for 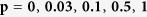 (black, green, yellow, red, blue), and *p*_1_ = 0.8, *p*_2_ = 0.2, *q*_1_ = 0.999, *q*_2_ = 0.9. The dashed lines correspond to the values calculated from 1000 realizations of the stochastic process. For a relatively high number of measurements *m* (>300) there is a clear monotonicity of 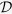 as a function of the degree of the noise time-correlations related to the quantity 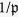.

Figure 4 shows the standard deviations 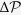 (i.e. the square root of the above calculated variances, but without normalization) together with numerical values from the realization of 1000 stochastic processes for each chosen value of 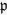. The underlying distribution *p*(Ω) is as in Fig. 3. We find that the larger is the time-correlation (the smaller 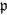), the larger is the standard deviation of the survival probability 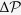, i.e. the more the outcome depends on the single realization. To average out the non-monotonic behaviour of 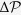, we consider the accumulated standard deviation


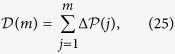


i.e. we sum up the standard deviation values for every measurement *j* = 1…*m*. The result is shown in Fig. 5. Indeed, for relatively large values of *m* (>300) 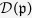 does monotonically increase with the amount of noise temporal correlations related to the quantity 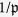.

## Discussion

If we compare the time-average with the ensemble one for different temperatures (i.e. 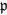), we find that the convergence of the time-average (quantified by the standard deviation) as well as the expected values for the ensemble average depend on 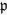. If we consider *N* realizations with *m* measurements each, then for large numbers *m* and *N* (i.e. many measurements and many realizations) the frequency of each event *q*(Ω) is *mNp*(Ω) independently of 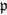. To calculate the time-average (for 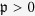) we will thus have *mp*(Ω) events and the value of the time-average is then 
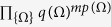
, as shown in Eq. (14). For the ensemble average, instead, we have to average over many realizations, where each time the exponent of *q*(Ω) will deviate from *mp*(Ω) according to the (possibly time-correlated) statistics. Because of these increasing deviations, the ensemble average for annealed disorder is larger than the time-average quantified in the Supplementary Information (SI). If we include time correlations, the ensemble average will grow until it takes the maximum for the quenched disorder limit, i.e. the arithmetic average of the quantity *q*(Ω)^*m*^. As a consequence, we find that


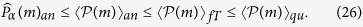


**Figure 6:**
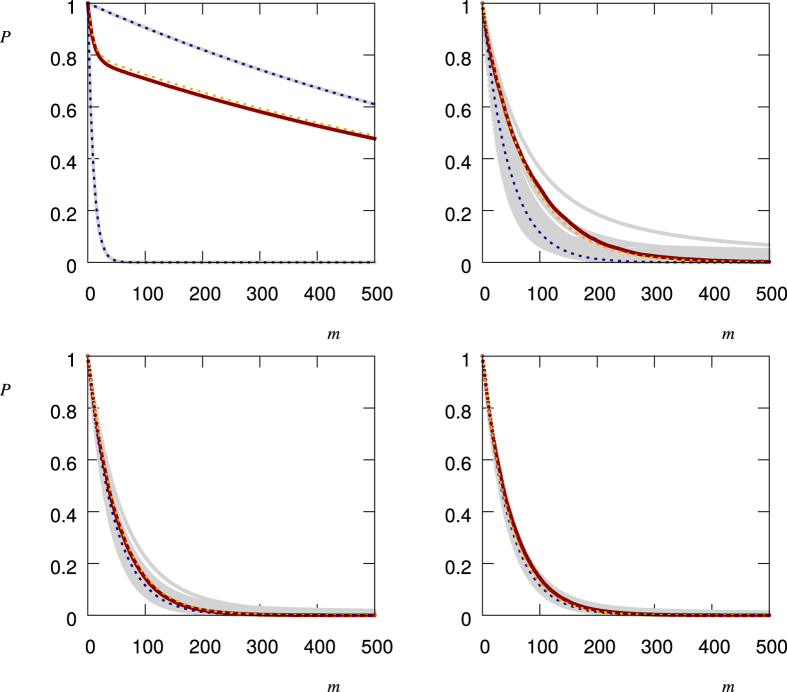
Ergodicity breaking. Numerical Values: 50 realizations of the time-average 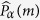, Eq. (4), with *M* = 2000 (grey solid lines), along with the ensemble average 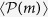, Eq. (5) calculated from 1000 realizations of the stochastic process (red solid lines), as a function of *m*. These are compared to the theoretical curves for the time-average (dark blue dashed) and ensemble average (orange dashed). Top left: quenched, top right: 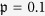, bottom left: 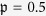, bottom right: annealed.

For annealed disorder the time and ensemble averages practically coincide: we refer to this equality as an ergodic property of the system-environment interaction^21^. However, the more the *q*(Ω_*I*,*α*_) are correlated, the more the ensemble average moves away from the time average and the ergodicity is broken. This can be seen in Fig. 6, where time and ensemble averages are simulated for a bimodal distribution *p*(Ω) for quenched and annealed disorder, and for two values of finite temperature. Also for this simulation, as well as for Figs 3, 4 and 5, we have used a bimodal distribution with *p*_1_ = 0.8, *p*_2_ = 0.2 and corresponding single measurement quantum survival probabilities *q*_1_ = 0.999, *q*_2_ = 0.9. As shown in the SI, the non-ergodic behavior depends essentially on the second and fourth moment of *p*(Ω). In other terms, for a similar average value, this effect will decrease if we choose *p*_1_ ≈ *p*_2_ or *q*_1_ ≈ *q*_2_. The same happens if we change the bimodal distribution into a multimodal or continuous distribution. From an application point of view, this allows us to detect correlations in a fluctuating field by measuring and comparing to each other the time and ensemble averages of the survival probability. Furthermore, by changing the time interval *μ* between two measurements, we can explore the time scale on which these correlations occur.

**Figure 7:**
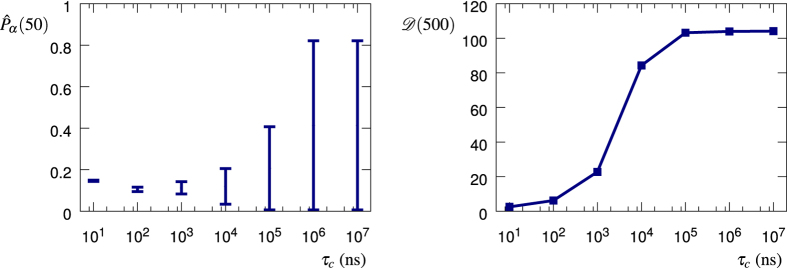
Convergence and relaxation time. The left panel shows how the convergence of the time-average 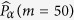, Eq. (4), for *M* = 2000 depends on the correlation time *τ*_*c*_. The error bars indicate the 20th and 80th percentiles and, thus, contain 30 final values for a total of 50 realizations of the time average calculated for *M* = 2000. The right panel shows how the accumulated standard deviation 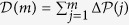, Eq. (25), depends on the correlation time *τ*_*c*_. We plot the value of 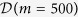 obtained from 1000 realizations of the stochastic process.

**Figure 8:**
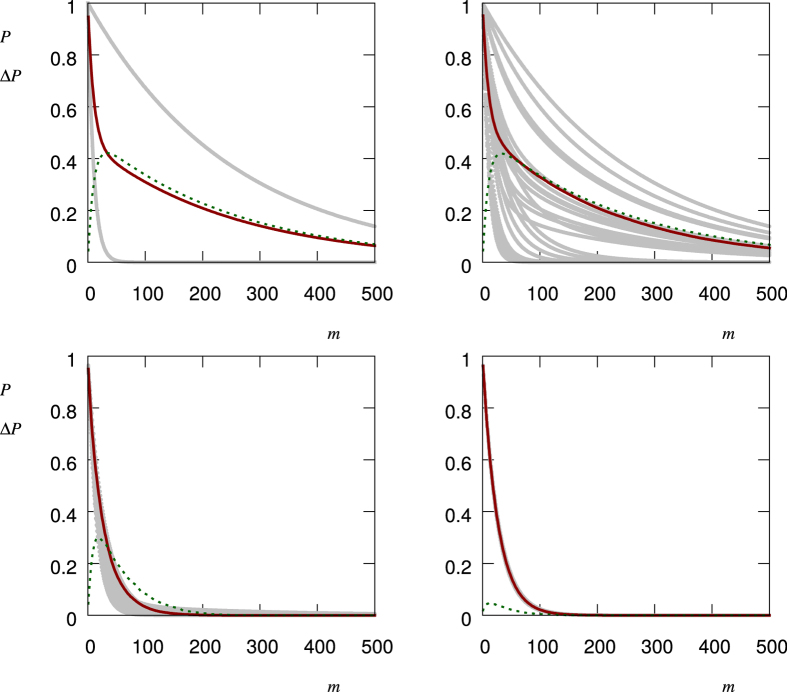
Time and ensemble averages, as in Fig. 6, along with the standard deviation 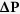 for the two-level Hamiltonian of Eq. 27 with a fluctuating RTN field. Numerical Values: 50 realizations of the time-average 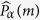, Eq. (4), (grey solid lines) with *M* = 2000, along with the ensemble average 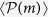, Eq. (5), calculated from 1000 realizations of the stochastic process (red solid line). The dark green dashed lines show the standard deviation 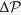 of the single realizations. The time scale *τ*_*c*_ of the correlation decreases from left to right and from top to bottom, ranging from perfectly correlated (quenched) disorder to uncorrelated (annealed) noise.

In order to effectively test our method for a real quantum system, we consider the two-level Hamiltonian


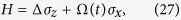


with the Pauli matrices *σ*_*x*_, *σ*_*z*_, a fluctuating driving Ω(*t*) of the system (e.g. an unstable classical light field) and a detuning term Δ. We set Δ = 2*π* × 5 MHz and Ω ∈ 2*π* × {1,5} MHz as a fluctuating RTN field with equal probability for both values. We initially prepare the system in the ground state |0〉 and perform projective measurements in this state spaced by intervals of constant length *μ* = 100 ns. Such scheme may be implemented on many different experimental platforms and, very recently, has been realized in the stochastic quantum Zeno context with a Bose-Einstein condensate on an atom-chip^20^ under similar conditions. Note that the second-order time correlation function for the RTN is exponential in time, i.e. 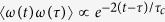, where the relaxation time *τ*_*c*_ is equal to the average time between two field switches. The left panel of Fig. 7 shows how the convergence of the time-average 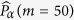 for *M* = 2000 depends on the correlation time *τ*_*c*_. The error bars indicate the 20th and 80th percentiles and, thus, contain 30 final values for a total of 50 realizations of the time average calculated for *M* = 2000. The right panel, instead, shows how the accumulated standard deviation 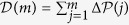, Eq. (25), depends on the correlation time *τ*_*c*_. We plot the value of 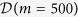 obtained from 1000 realizations of the stochastic process. Finally, Fig. 8 shows the time and ensemble averages along with the standard deviation, for an average time between the fluctuating field switches such as 10,10^3^,10^5^,10^7^ ns. It can be clearly seen how a noise correlation time longer than the time interval *μ* generates a growing standard deviation of the survival probability, which can then be exploited as a witness of time-correlated noise.

## Conclusions

By studying SQZD in time-correlated environments we have shown how an ergodicity property quantitatively depends on the time scale of the noise correlations. By doing so, we propose a new (quantum Zeno-based) way to detect time correlations in random classical fields coupled to a quantum probing system. The time correlations in the noise field determine whether and how fast the survival probability converges to its statistical mean, hence how the standard deviation of the survival probability over many experimental realizations will reveal information on the noise field. Then, we further improve this dependence by summing the standard deviation over the whole measurement series. This way we obtain a witness of time correlations in the fluctuating field coupled to the quantum probe. Let us stress that this approach can be generalized by applying different measurement operators. Indeed, it has been demonstrated that quantum Zeno dynamics allows to confine the dynamics within decoherence-free subspaces^38^,^39^. By turning around that point of view, one can realize different initial states and measurement operators and thus also probe the effect of the environment on different subspaces of the system. Each of them might experience different time correlations of the noise as they predominantly couple to a different bandwidth of the noise spectrum. In conclusion we have introduced a novel method to examine time correlations and spectra of an environment acting on a quantum probing system as a random Hamiltonian term. This method does not rely on quantum state and process tomography but on a simple Zeno-based measurement scheme. It is also platform independent and thus can be used in very different implementations of the physical system and frequency/time scale ranges of the noisy environment field. Therefore, these results are expected to move further steps towards novel technologies for quantum sensing, where the fragile properties of quantum systems, as coherence, and especially here Zeno phenomena are exploited to probe an environmental fluctuating field and indirectly the presence of external artificial and biological molecules that are difficult to image otherwise.

## Additional Information

**How to cite this article**: Müller, M. M. *et al*. Stochastic quantum Zeno-based detection of noise correlations. *Sci. Rep.* **6**, 38650; doi: 10.1038/srep38650 (2016).

**Publisher's note:** Springer Nature remains neutral with regard to jurisdictional claims in published maps and institutional affiliations.

## Supplementary Material

Supplementary Information
