# Supplementary material for: Stochastic quantum Zeno-based detection of noise correlations
Source: Sci Rep. 2016 Dec 12;6:38650. doi: 10.1038/srep38650 (PMC5150251; doi:10.1038/srep38650)
Supplement: Supplementary Information [file srep38650-s1.pdf]

Supplementary Information: Stochastic quantum Zeno-based detection of noise correlations

Matthias M. Müller[1], Stefano Gherardini[1,2], and Filippo Caruso[1,*]

[1]Department of Physics and Astronomy and LENS, University of Florence, via G. Sansone 1, I-50019 Sesto Fiorentino, Italy, and QSTAR, Largo E. Fermi 2, I-50125 Florence, Italy.
[2]Department of Information Engineering, University of Florence, via S. Marta 3, I-50139 Florence, Italy & CSDC, University of Florence, and INFN, via G. Sansone 1, I-50019 Sesto Fiorentino, Italy.
[*]Corresponding author: filippo.caruso@lens.unifi.it

ABSTRACT

A system under constant observation is practically freezed to the measurement subspace. If the system driving is a random classical field, the survival probability of the system in the subspace becomes a random variable described by the Stochastic Quantum Zeno Dynamics (SQZD) formalism. Here, we study the time and ensemble average of this random survival probability and demonstrate how time correlations in the noisy environment determine whether the two averages do coincide or not. These environment time correlations can potentially generate non-Markovian dynamics of the quantum system depending on the structure and energy scale of the system Hamiltonian. We thus propose a way to detect time correlations of the environment by coupling a quantum probe system to it and observing the survival probability of the quantum probe in a measurement subspace. This will further contribute to the development of new schemes for quantum sensing technologies, where nanodevices may be exploited to image external structures or biological molecules via the surface field they generate.

Quantum Zeno regime

If the time interval between two measurements μ is small compared to the system dynamics ($\Delta^2 H\mu^2 \ll 1$) and we assume $H_0 = 0$, we can approximate $q(\tilde{\Omega})$ by a Taylor expansion in $\tilde{\Omega}^2\mu^2$, where $\tilde{\Omega} = \frac{1}{\mu}\int_0^{\mu}\Omega(t)dt$ is again the mean value of the field within the time interval. For simplicity, from now on we omit the tilde symbol and use $q(\Omega)$ and Ω. Then, we consider also the Taylor expansion for the survival probability of the measurement sequence (note that this requires the stricter Zeno condition $m\Delta^2 H\mu^2 \ll 1$). This allows us to analytically quantify the discrepancy between the different averages in the Zeno regime. In particular, we analyze the two extreme cases, i.e. the time average for annealed disorder and the ensemble average for quenched disorder. Starting from Eq. (22) in the Main, we decrease the time interval μ such that we enter into the quantum Zeno dynamics regime. Then all time and ensemble averages collapse to one value (different from 1, though), as theoretically demonstrated and experimentally observed in Ref.[1]. This approximation error is given by the quantity:

$$\frac{\langle \mathscr{P}(m)\rangle_{qu} - \hat{P}_{\alpha}(m)_{an}}{\hat{P}_{\alpha}(m)_{an}} = \exp\left\{\ln\langle q(\Omega)^m\rangle - \langle \ln q(\Omega)^m\rangle\right\} - 1 \approx \Delta q \ ,$$

with

$$\Delta q = \ln\langle q(\Omega)^m\rangle - \langle \ln q(\Omega)^m\rangle = \frac{m^2}{2}(\Delta^2 H_{noise})^2(\omega_4 - \omega_2^2)\mu^4 \ ,$$

with ω_2 and ω_4 the second and third moment of the distribution $p(\Omega)$. As a consequence, when we approach the Zeno limit all the averages collapse to the same value. Note that this is not trivial since the convergence is to the fourth order of the time interval while the leading order of the averages is the second one. The parameter Δq can thus be considered to be the parameter that drives the transition from ergodic behavior to non-ergodic one, where in the strict Zeno regime all the effects of time correlations vanish regardless of the temperature. Instead, when we increase Δq by moving out of the Zeno regime, temperature-dependent correlation effects cause a splitting of the values for the ensemble average of the survival probability.

Concerning the variance of the distribution $Prob(\mathscr{P})$, in the special case of infinite temperature or annealed disorder, it is given by

$$\langle \mathscr{P}^2(m)\rangle_{an} = \int d\mathscr{P} Prob(\mathscr{P})\mathscr{P}^2 = \int d\Omega_1 \ldots \int d\Omega_m \prod_{i=1}^{m} p(\Omega_i)q(\Omega_i)^2 = \exp\left\{m\ln\langle q(\Omega)^2\rangle\right\} \ . \quad (1)$$

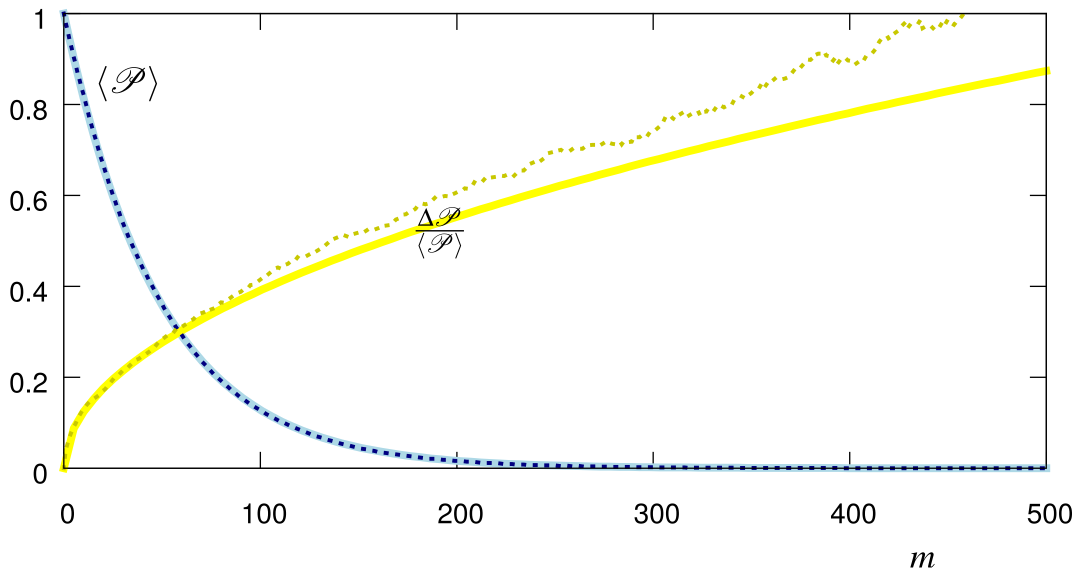

Figure 1. Ensemble average for 1000 realizations of a stochastic sequence with annealed disorder ($T=\infty$). $\langle\mathscr{P}(m)\rangle$ (simulation dark-blue, theory light-blue) and normalized standard deviation $\Delta\mathscr{P}(m)/\langle\mathscr{P}(m)\rangle$ (simulation dark-yellow, theory light-yellow) are shown as a function of m .

The normalized variance thus reads

$$\frac{\Delta^2\mathscr{P}(m)_{an}}{\langle\mathscr{P}(m)\rangle_{an}^2}=\frac{\langle\mathscr{P}(m)^2\rangle_{an}-\langle\mathscr{P}(m)\rangle_{an}^2}{\langle\mathscr{P}(m)\rangle_{an}^2}=\exp\left\{m\left[\ln\langle q(\Omega)^2\rangle-\ln\langle q(\Omega)\rangle^2\right]\right\}-1\approx m\left(\ln\langle q(\Omega)^2\rangle-\ln\langle q(\Omega)\rangle^2\right), \tag{2}$$

which leads to the normalized standard deviation

$$\frac{\Delta\mathscr{P}(m)_{an}}{\langle\mathscr{P}(m)\rangle_{an}}\approx\sqrt{m}\sqrt{\ln\langle q(\Omega)^2\rangle-\ln\langle q(\Omega)\rangle^2}\approx\sqrt{m}\Delta^2H_{noise}\mu^2\sqrt{\omega_4-\omega_2^2}, \tag{3}$$

where the latter expression is a second-order expansion in the interval length, and ω_2 and ω_4 are the second and fourth statistical moments of $p(\Omega)$. Fig. 1 shows the ensemble average along with the normalized standard deviation for annealed disorder as a function of m.

For the finite-temperature case, again we first consider a sequence of constant Ω. The square of the survival probability is given by

$$\langle\mathscr{P}_{\mathfrak{p}}^2\rangle=\sum_{k=0}^{\infty}r_\lambda(k)\int p(\Omega)(q(\Omega)^2)^k d\Omega=\int p(\Omega)\mathrm{e}^{\frac{q(\Omega)^2-1}{\mathfrak{p}}}d\Omega. \tag{4}$$

The frequency of the field updates is again Poisson distributed, with expectation value $\mathfrak{p}m$. The joint squared survival probability is then

$$\langle\mathscr{P}(m,\mathfrak{p})^2\rangle_{fT}=\mathrm{e}^{-\mathfrak{p}m}\sum_{k=0}^{\infty}\frac{(\mathfrak{p}m)^k}{k!}\langle\mathscr{P}_{\mathfrak{p}}^2\rangle^k=\exp\left\{\mathfrak{p}m(\langle\mathscr{P}_{\mathfrak{p}}^2\rangle-1)\right\}. \tag{5}$$

The normalized variance thus reads

$$\frac{\Delta^2\mathscr{P}(m,\mathfrak{p})_{fT}}{\langle\mathscr{P}(m,\mathfrak{p})\rangle_{fT}^2}=\frac{\langle\mathscr{P}(m,\mathfrak{p})^2\rangle_{fT}-\langle\mathscr{P}(m,\mathfrak{p})\rangle_{fT}^2}{\langle\mathscr{P}(m,\mathfrak{p})\rangle^2}\approx m\left[\frac{1}{\mathfrak{p}}+1\right](\Delta^2H_{noise})^2\mu^4\omega_4, \tag{6}$$

which leads to the normalized standard deviation

$$\frac{\Delta\mathscr{P}(m,\mathfrak{p})_{fT}}{\langle\mathscr{P}(m,\mathfrak{p})\rangle_{fT}}\approx\sqrt{m}\sqrt{1+\frac{1}{\mathfrak{p}}}\Delta^2H_{noise}\mu^2\sqrt{\omega_4}. \tag{7}$$

For quenched disorder, one gets

$$\langle\mathscr{P}^2(m)\rangle_{qu}=\int d\mathscr{P}P(\mathscr{P})\mathscr{P}^2=\int d\Omega p(\Omega)q(\Omega)^{2m}=\exp\left\{\ln\langle q(\Omega)^{2m}\rangle\right\}. \tag{8}$$

The normalized variance then reads

$$\frac{\Delta^2 \mathscr{P}(m)_{qu}}{\langle \mathscr{P}(m)\rangle_{qu}^2} = \frac{\langle \mathscr{P}(m)^2\rangle_{qu} - \langle \mathscr{P}(m)\rangle_{qu}^2}{\langle \mathscr{P}(m)\rangle_{qu}^2} = \exp\left\{\left[\ln\langle q(\Omega)^{2m}\rangle - \ln\langle q(\Omega)^m\rangle^2\right]\right\} - 1 \approx \left(\ln\langle q(\Omega)^{2m}\rangle - \ln\langle q(\Omega)^m\rangle^2\right), \quad (9)$$

and the normalized standard deviation is thus

$$\frac{\Delta \mathscr{P}(m)_{qu}}{\langle \mathscr{P}(m)\rangle_{qu}} \approx \sqrt{\ln\langle q(\Omega)^{2m}\rangle - \ln\langle q(\Omega)^m\rangle^2} \approx m\Delta^2 H_{noise}\mu^2\sqrt{\omega_4 - \omega_2^2}\,. \quad (10)$$

where the latter expression is a second-order expansion in the time interval length.

References

1. Gherardini, S. *et al.* *Eprint arXiv:1604.08518* .
